# Melatonin Application Alleviates Stress-Induced Photosynthetic Inhibition and Oxidative Damage by Regulating Antioxidant Defense System of Maize: A Meta-Analysis

**DOI:** 10.3390/antiox11030512

**Published:** 2022-03-08

**Authors:** Ihsan Muhammad, Li Yang, Shakeel Ahmad, Ibrahim S. M. Mosaad, Abdullah Ahmed Al-Ghamdi, Arshad Mehmood Abbasi, Xun-Bo Zhou

**Affiliations:** 1 Guangxi Colleges and Universities Key Laboratory of Crop Cultivation and Tillage, Agricultural College, Guangxi University, Nanning 530004, China; ihsan@stumail.nwu.edu.cn (I.M.); yangli0331@st.gxu.edu.cn (L.Y.); shakeel@gxu.edu.cn (S.A.); 2 Soil Fertility and Plant Nutrition Research Department, Soil, Water and Environment Research Institute, Agricultural Research Center, Giza 12619, Egypt; ibrahim.mosad@arc.sci.eg; 3 Department of Botany and Microbiology, College of Science, King Saud University, P.O. Box 2455, Riyadh 11451, Saudi Arabia; abdaalghamdi@ksu.edu.sa; 4 Department of Environmental Sciences, COMSATS University Islamabad, Abbottabad Campus 22060, Pakistan; amabbasi@cuiatd.edu.pk or; 5 University of Gastronomic Sciences, 12042 Pollenzo, Italy

**Keywords:** maize varieties, melatonin, heavy metals, environmental stress, meta-analysis, heterogeneity

## Abstract

Melatonin is effective in modulating metabolism and regulating growth and development in many plants under biotic and abiotic stress. However, there is no systematic quantification of melatonin effects on maize growth, gas exchange, chlorophyll content, and the antioxidant defense system. A meta-analysis was conducted on thirty-two currently available published articles to evaluate the effect of stress types, study types, and maize varieties on response ratio (ln*RR*_++_) of “melatonin” to “control (no melatonin)” on plant growth, enzyme activities, gas exchange parameters, and photosynthetic pigments. Our findings revealed that melatonin application overall increased plant height, leaf area, root length, fresh and dry root weight and shoot weight, superoxide dismutase (SOD), peroxide (POD), catalase (CAT), ascorbate peroxidase (APX), soluble sugar and protein, photosynthetic rate, stomatal conductance, transpiration rate, chlorophyll, and carotenoid in maize leaf under stress conditions. In contrast, melatonin application decreased the levels of hydrogen peroxide (H_2_O_2_), superoxide anion (O_2^−^_), malondialdehyde (MDA), and electrolyte leakage. The categorical meta-analysis demonstrated that melatonin application to chilling stress resulted in higher SOD activity followed by salt stress. Melatonin application to all stress types resulted in higher POD, CAT and APX activities, except Cd stress, which had no effect on POD and decreased CAT by 38% compared to control. Compared to control, melatonin resulted in lower reactive oxygen species (ROS) and electrolyte leakage under no stress, Cd, drought, salt, lead, heat, and chilling stress in all study types (pot, growth chamber, hydroponic, and field), except O_2_ content which was not affected in pot and growth chamber studies. It was concluded that melatonin alleviates oxidative damage by improving stress tolerance, regulating the antioxidant defense system, and increasing leaf chlorophyll content compared to control.

## 1. Introduction

Maize (*Zea mays* L.) is one of the most important crops in the world and is cultivated extensively as a staple food in almost every part of the world under a variety of environmental conditions [1,2]. It is a multipurpose crop due to its nutritional value, and it is used as food for humans, as feed for animals and poultry, and as an important raw material for industries around the world [1,3]. Scientists are most concerned about maize because it can survive in harsh environments [4]. However, adverse environmental conditions and heavy metal accumulation caused by human activity may limit its growth and productivity [5,6]. It goes without saying that plants, with their complex physiology, will inevitably find life challenging in environments with variable stress conditions, such as varied biotic and abiotic stress factors. Salinity, heavy metal pollution, drought, heat, and chilling stress are the most serious threats to plant growth, productivity, and human health in the agro-ecosystem, particularly in areas with high anthropogenic heaviness [7,8]. 

Heavy metal pollution, drought, salinity, lead (Pb), heat, and chilling stress are the primary threats to plant growth and crop yield and are increasingly being recognized as a serious issue throughout the world [1,3,9,10,11]. Cadmium is one of the most hazardous metals for a variety of food crops, particularly maize, and it has been shown to limit crop growth by disrupting normal cellular activities and metabolism [1,12]. Changes in morpho-physiological development and antioxidant enzyme activities in maize caused by cadmium also led to oxidative stress in maize due to an increase in reactive oxygen species generation (ROS) [1,13]. Moreover, it was observed that Cd toxicity decreased maize photosynthetic pigments, antioxidant enzyme activity, and morphological growth [14]. Maize is an essential crop in agricultural production, but the soil in China is currently polluted by Cd in many regions, leading to the enhancement of Cd content [15]. Consequently, it is imperative that scientists figure out how to reduce the amount of Cd in crops grown in polluted areas. 

The production of maize in arid and semi-arid areas of China is wholly reliant on rainfall. However, extremely limited and unpredictable precipitation during the growing period in these areas causes drought stress, which dramatically reduces maize production [16,17]. Moreover, drought is a major abiotic stressor that significantly negatively impacts corn production, resulting in a 40% decrease in maize yield globally over the last 25 years [2,18]. Even still, fertilizers and plant growth regulators are critical for increasing crop yield [16,19,20]. In agricultural production, drought or osmotic stress-induced water deprivation is one of the most severe abiotic stressors. On the other hand, melatonin has been widely reported to boost plant tolerance to water deprivation stress in a variety of plants [21,22]. 

Likewise, salinity is also a critical abiotic factor that affects crop growth and development [23,24]. Around the world, more than 0.8 billion hectares of farmland are severely salinized, making it a serious agricultural concern that results in low productivity [24,25]. Several essential metabolic processes in plants, including glucose metabolism, lipid metabolism, and protein synthesis, are adversely affected by salinity to a great extent because of ionic and osmotic stress [24,26,27]. Several ways have been used to maintain osmotic homeostasis and ion balance and avoid damage to plants to mitigate the detrimental effects of salinity stress [28,29,30,31]. Salinity stress also causes a decrease in chlorophyll content and stomatal conductance, which diminishes carbon dioxide assimilation, damage to photosynthetic organs, nutritional imbalance, oxidative damage, metabolic disorders, and alterations in the photosynthesis-related enzyme activity [31,32]. According to the findings of Ahanger et al. [33], salt stress decreased chlorophyll content, stomatal conductance, net photosynthesis, and transpiration in tomatoes. In recent years, several plant growth regulators have been employed to ameliorate the negative effects of stress on plants, such as salicylic acid [34], proline [35], nitric oxide [36] and melatonin [30]. External treatments are among the most appropriate and cost-effective techniques to boost germination and eventual yields under various abiotic stressors [30]. Melatonin is required for plant growth, germination, and development, and it also increases plant resistance to abiotic stresses such as salt stress, drought stress, heat stress, and cold stress conditions [30,37].

Application of melatonin enhances plant growth and photosynthetic activity, increases chlorophyll content, and decreases ROS formation and hence oxidative damage to plants [38]. Ren et al. [39] reported that melatonin application to stressed plants significantly increases leaf area, biomass, and photosynthetic efficiency. Additionally, melatonin application in saline circumstances decreased the Na^+^ level of the root and leaf and raised the K^+^ content [40]. As a result, improving maize plants’ stress tolerance is critical for increasing maize yields. Although melatonin is increasingly being used to improve plant growth, enzymatic activities, gas exchange parameters, and photosynthetic pigments [30,41,42], no meta-analysis comparing different stress types, study types, and maize varieties has been reported. In order to better understand the effects of exogenous melatonin dosages on maize seedling and growth performance, photosynthetic activity, stress sensitivity index, and enzymatic activities, a meta-analysis based on the available literature was conducted to synthesize the overall effect of melatonin on plant growth, gas exchange parameters, photosynthetic pigments, and enzymatic activities under different stress types, study types, and maize varieties. In this meta-analysis, we hypothesized that (1) melatonin application would have an overall positive effect on plant growth and enzyme activities, (2) such an effect would vary with stress types, study types, and maize varieties, and (3) optimum melatonin application would improve plant growth and enzyme activities that reduce oxidative plant damages.

## 2. Materials and Methods

### 2.1. Database Construction and Literature Search

The goal of this meta-analysis is to quantify the effect of melatonin on maize plant growth, gas exchange (net photosynthetic rate, intercellular CO_2_, stomatal conductance, transpiration rate), chlorophyll content, and improving enzymatic and non-enzymatic activities. A total of 32 studies across the globe were collected from the available literature on the Web of Science (www.sciencedirect.com; accessed on 18 May 2021) for this meta-analysis. Keywords, such as net photosynthetic rate, intercellular CO_2_, stomatal conductance, transpiration rate, enzyme activities, chlorophyll content, heavy metal stress (cadmium and lead stress), drought stress, salt stress, chilling stress, antioxidant defense system, and melatonin, were used for the search. Experiment and stress types, such as temperature, humidity, light intensity, and maize verity of the selected studies were also recorded. Meta-analyses, unlike traditional literature reviews, are led by a specific search procedure that contains defined inclusion and exclusion criteria as well as a screening process that further determines study eligibility.

The data for the above-mentioned parameter was taken from the literature. Graph digitizer software GetData 2.26 was used to extract the data presented in graphical form. All kinds of studies (field study, growth chamber, and greenhouse experiment) were included for the data collection on melatonin with control (no melatonin). The PRISMA statement (Preferred Reporting Items for Systematic Reviews and Meta-Analyses, http://www.prisma-statement.org/ accessed on 21 December 2021) was employed for reporting the screening process. After removing duplicate records, all the publications were checked against a set of exclusion and inclusion criteria Studies were included having three or above replications and standard error (SE) or standard deviation (SD), while discarded with no true control of melatonin [43]. Data was collected from publications comparing melatonin with no melatonin under the maize crop on plant gas exchange parameters, chlorophyll, and enzymatic and non-enzymatic activities. The published studies were discorded from the database with the lack of mean value, SD, SE and replication. The standard deviation of the mean was calculated through the following equation:

(1) $$\mathrm{SD}=\mathrm{SE}\;\times \sqrt{n}$$

whereas n is the number of replications, SD and SE are the standard deviations and standard error of the mean. The current meta-analysis, including 1649 pairwise comparisons was drawn from 32 published studies that met the aforementioned criteria. In order to evaluate the performance of melatonin and control, we collected the quantitative data and analyzed the difference.

### 2.2. Data Calculation and Analysis

The effect of melatonin on maize growth, enzymatic activities, and gas exchange parameters were estimated through response ratios (RRs) as the effect size using MetaWin software. Melatonin was tested on measured parameters using the RR [44,45], and was calculated using the following equation:

(2) $$\mathrm{RRs}=\mathrm{Ln}\left(\frac{X_{M}}{X_{\mathrm{NM}}}\right)=\mathrm{Ln}\left(X_{M}\right)-\mathrm{Ln}\left(X_{\mathrm{NM}}\right)$$

whereas X_M_ and X_NM_ are the means of enzymatic activities, plant growth, and gas exchange parameters in melatonin and control treatment. The natural log ratio is used to confirm that the numerator and denominator changed proportionately. Different stress types, maize varieties, and experimental types were considered distinct observations for each study. The variance of RRs for each sample was calculated as follows.

(3) $$V=\frac{S_{M}^{2}}{n_{M}{\;X}_{M}^{2}}+\frac{S_{\mathrm{NM}}^{2}}{n_{\mathrm{NM}}{\;X}_{\mathrm{NM}}^{2}}\;$$

where S_M_ and S_NM_ are the standard deviations, n_M_ and n_NM_ are the replication of the studies, and X_M_ and X_NM_ are the mean values of melatonin and control treatments, respectively. The weight (W) for each RRs was calculated through the following equation:

(4) W=1V

The overall mean response ratio (RR_E++_) for individual melatonin treatment was computed using the equation as follows:

(5) $${\mathrm{RR}}_{E++}=\frac{\sum_{i=1}^{n}\sum_{j=1}^{m}W_{\mathrm{ij}}{\mathrm{RR}}_{\mathrm{ij}}}{\sum_{i=1}^{n}\sum_{j=1}^{m}W_{\mathrm{ij}}}\;$$

where “n” and “m” are the number of treatments and comparisons of the enzyme’s activities, maize growth, and gas exchange parameters, respectively. The SE of overall response ratio (RR_E++_) was calculated as:

(6) $$\mathrm{SE}\left({\mathrm{RR}}_{E++}\right)=\sqrt{\frac{1}{\sum_{i=1}^{n}\sum_{j=1}^{m}W_{\mathrm{ij}}}}\;$$

To perform a meta-analysis and evaluate the effect of melatonin on enzymes, maize growth, and gas exchange parameters by computing the mean effect size and 95% bootstrapped confidence intervals (CIs), the metaWin 2.1 was used [43,45]. Melatonin was considered to be significant if the 95% confidence intervals did not overlap the zero line. The effect of melatonin on stress types, study types, and maize varieties were also examined among the selected parameters. The total heterogeneity of ln*RR*_++_ (95% CIs) among studies (Q_T_) was calculated using categorical analysis in MetaWin, and then the I-square index was calculated by dividing the difference between Q_T_ and degrees of freedom (n − 1) by Q_T_ [45,46]. Table S1, Figures S1–S3 in the Supplementary Information summarizes the data heterogeneities for the target variables, with greater Q_T_ and I^2^ values indicating a significant amount of heterogeneity [45]. Rosenthal’s failsafe number and Spearman rank-order correlation were used for publication bias in MetaWin 2.1, with a failsafe number greater than 5n + 10 indicating no publication bias (Table S1), where n is the observation [47,48]. The heterogeneity of this meta-analysis was calculated as:

(7) $$Q=\overset{k}{\underset{i=1}{\sum }}W_{i}{\left(\mathrm{In}R_{i}\right)}^{2}-\frac{{\left(\sum_{i=1}^{k}W_{i}\mathrm{In}R_{i}\right)}^{2}}{\sum_{i=1}^{k}W_{i}}$$

Additionally, we used Origin software 2021 to plot the kernel density estimations for enzyme activities, maize growth, and gas exchange parameters.

## 3. Results

### 3.1. Publication Bias and Data Heterogeneity

Thirty-two published studies with a total of 1649 pairwise observations were available for meta-analysis, and most of these studies involved multiple cases. Our meta-analysis indicated that data were normally distributed with strong heterogeneity for all moderators (fresh and dry root weight, fresh and dry shoot weight, plant height, leaf area, root length and diameter, photosynthetic rate, stomatal conductance, transpiration rate, intercellular CO_2_ concentration, chlorophyll a and b, carotenoid and total chlorophyll content, relative water content, SOD, POD, CAT, APX, GPX, O_2_, H_2_O_2_, MDA, proline, soluble sugar, soluble protein and electro leakage) among stress types, study types, and maize varieties, as characterized with greater values of Q_T_ and I-square (Figures S1–S3 and Table S1). The greater fail-safe numbers for all the above-mentioned parameters indicate no publication bias (Table S1), suggesting that the quality of the study meets the standard for meta-analysis.

### 3.2. Exogenous Melatonin Improved Biomass and Plant Growth 

**Figure 1:**
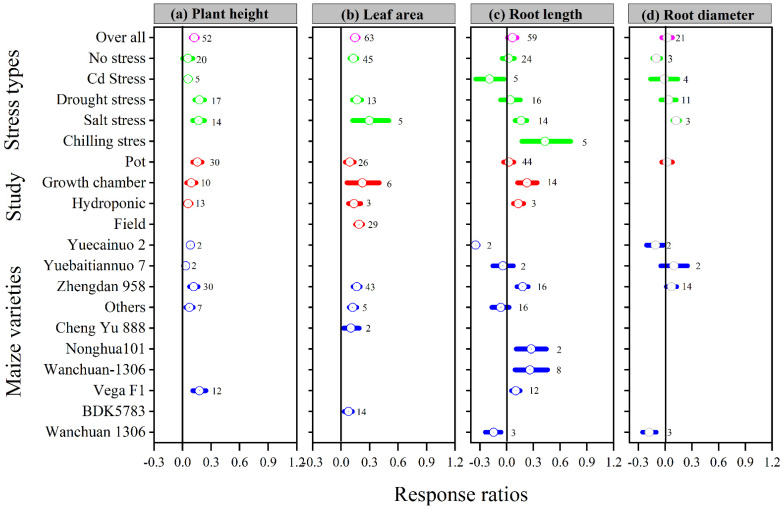
Response ratio of melatonin compared to control with bootstrapped 95% confidence interval on plant height (**a**), leaf area (**b**), root length (**c**), and root diameter (**d**), for different stress types, study types, and maize varieties. The vertical line (ln*RR*_++_ = 0) indicates no difference between melatonin and control. Numbers following the box indicate the number of observations for comparison.

The overall ln*RR*_++_ was positive for plant height, leaf area, and root length with melatonin application; however, the overall ln*RR*_++_ for root diameter was not affected (Figure 1). These results suggest that the melatonin application increased plant height, leaf area, and root length compared to control. The categorical meta-analysis showed that different stress types, study types, and maize varieties have a variable effect on the above-mentioned parameter. Melatonin application significantly increased plant height and leaf area with all stress types, study types, and maize varieties, except Cd stress for plant height. Among the stress types, drought stress had the maximum plant height, which is not statistically different from salt stress. The categorical analysis also revealed that among the study types, the pot study had significantly higher plant height than hydroponic, while the maximum leaf area was observed for field study, which is significantly higher than the pot study (Figure 1). Among the maize varieties, Yuecainuo 2 and Wanchuan 1306 resulted in lower ln*RR*_++_ for root length and Wanchuan 1306 for root diameter, however, the Zhengdan 958 had positive and greater ln*RR*_++_ for both root length and root diameter.

**Figure 2:**
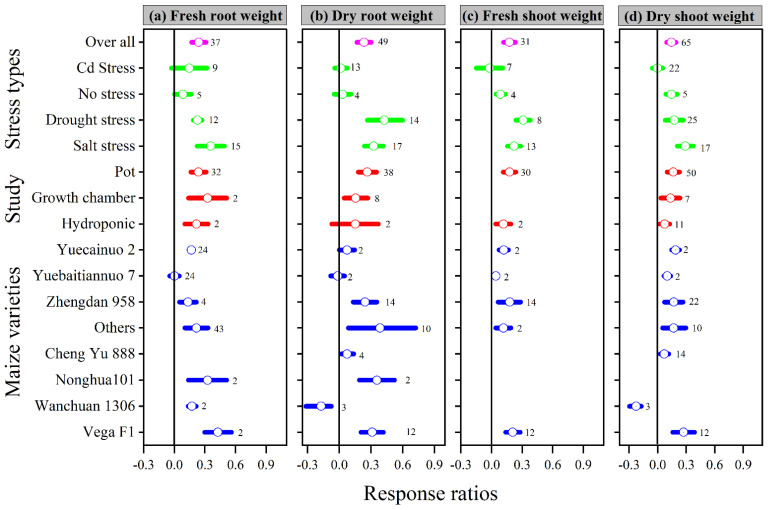
Response ratio of melatonin compared to control with bootstrapped 95% confidence interval on fresh root weight (**a**), dry root weight (**b**), fresh shoot weight (**c**), and dry shoot weight (**d**), for different stress types, study types, and maize varieties. The vertical line (ln*RR*_++_ = 0) indicates no difference between melatonin and control. Numbers following the box indicate the number of observations for comparison.

**Figure 3:**
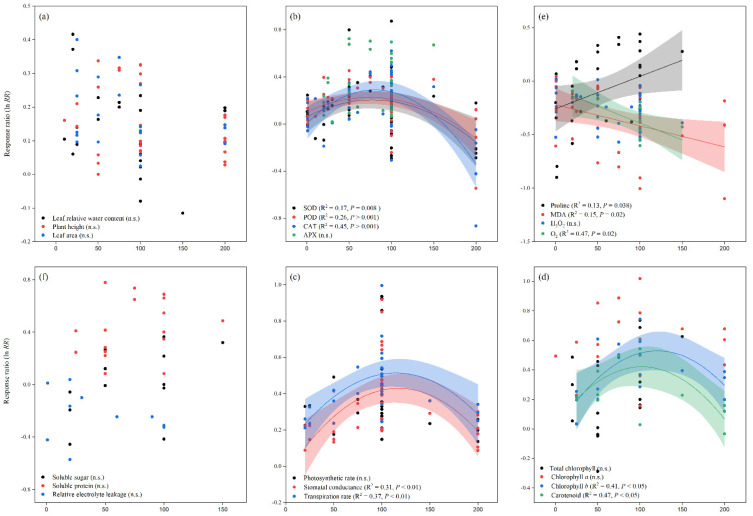
Relationships between the response ratios of melatonin compared to control, leaf relative water content, plant height, and leaf area (**a**), SOD, POD, CAT, and APX (**b**), proline, MDA, H_2_O_2_ and O_2_ (**c**), soluble sugar, soluble protein, and relative electrolyte leakage (**d**), photosynthetic rate, stomatal conductance, and transpiration rate (**e**), total chlorophyll, chlorophyll *a,* chlorophyll *b*, and carotenoid (**f**). The horizontal dash line (ln*RR*_++_ = 0) indicates no difference between cover cropping and no cover cropping systems.

Similarly, when compared to the control, melatonin application increased the overall ln*RR*_++_ of fresh and dry root weight, as well as fresh and dry shoot weight, by 24, 23, 18, and 14%, respectively. The ln*RR*_++_ of fresh and dry root weight and fresh and dry shoot weight to melatonin application differed among stress types, study types, and maize varieties (Figure 2). Melatonin application significantly increased fresh and dry root weight under drought and salt stress but had no significant effect under Cd and no stress conditions. Fresh and dry shoot weight was significantly and positively affected by melatonin application with all stress types except Cd stress. Among the stress types, drought and salt stress had greater ln*RR*_++_ for fresh shoot weight and dry shoot weight, respectively. Among the study types, the growth chamber had the highest ln*RR*_++_ for fresh root weight and pot study for dry root weight, fresh and dry shoot weight (Figure 2). In contrast, no significant effect of melatonin application on dry root weight was observed for hydroponic study. The categorical analysis for maize varieties suggests that most of the varieties resulted in positive ln*RR*_++_, however, Wanchuan 1306 had negative ln*RR*_++_ for dry root and dry shoot weight. These results suggest that melatonin application to the maize variety Wanchuan 1306 decreased the dry root weight by 32 and the dry shoot weight by 30% compared to control. The regression analysis showed that the response ratio of plant height and leaf area was not related to melatonin application (Figure 3a).

### 3.3. Exogenous Melatonin Improved Enzymatic Activities

**Figure 4:**
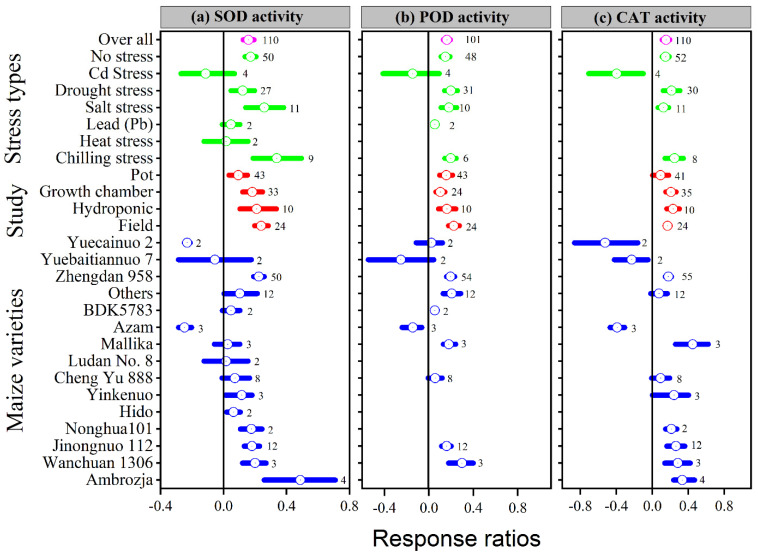
Response ratio of melatonin compared to control with bootstrapped 95% confidence interval on SOD (**a**), POD (**b**), and CAT activities (**c**), for different stress types, study types, and maize varieties. The vertical line (ln*RR*_++_ = 0) indicates no difference between melatonin and control. Numbers following the box indicate the number of observations for comparison.

**Figure 5:**
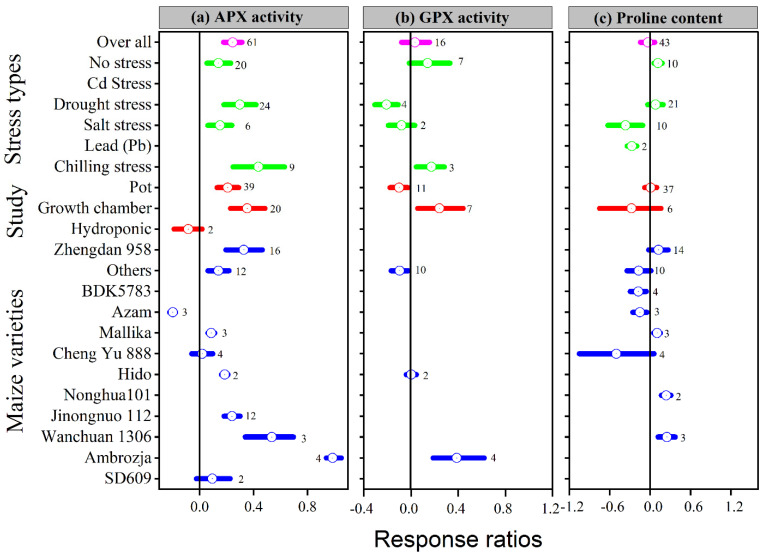
Response ratio of melatonin compared to control with bootstrapped 95% confidence interval on APX (**a**), GPX (**b**), and proline content (**c**), for different stress types, study types, and maize varieties. The vertical line (ln*RR*_++_ = 0) indicates no difference between melatonin and control. Numbers following the box indicate the number of observations for comparison.

Application of melatonin increased overall antioxidant enzyme activities (SOD, POD, CAT and APX) by 16, 16, 15 and 24%, respectively, compared to control (*p* ≤ 0.05, Figure 4a–c and Figure 5a). In contrast, no significant effect of melatonin application on GPX activity was observed for the overall effect (Figure 5b). The categorical analysis showed that the melatonin application to Cd, lead, and heat stress had no effect on SOD activity. These results suggest that the ln*RR*_++_ for Cd, lead and heat stress overlaps the zero line, which means melatonin had no significant effect on SOD activity compared with control. Chilling stress resulted in higher SOD activity compared to no stress, drought stress, and salt stress; however, this increase was statistically similar. 

Our results showed that the effect of melatonin application on POD, CAT and APX activities was significantly higher and positive with all types of stresses (Figure 4b,c and Figure 5a), except Cd stress for POD and CAT activities. Similarly, the ln*RR*_++_ of melatonin for SOD and POD activities were significantly higher in all study types, with a significantly higher increase of 24 and 22% in the field study (Figure 4a,b). In contrast, there was no significant effect of melatonin on CAT activities in the pot study over control (Figure 4c), however, the pot study significantly decreased the GPX activity (Figure 5b). The ln*RR*_++_ of melatonin application on enzymatic activities also varied with different maize varieties (Figure 4 and Figure 5). The ln*RR*_++_ of SOD activity was significantly greater in the Ambrozja variety than in the Hido variety, but not significantly different from Zhengda 958, others, Yinkenuo, Nonghua 101, Jinonguo 112, and Wanchuan 1306. Among the maize varieties, the ln*RR*_++_ of Yuecainuo, Yuebaitiannuo, and Cheng Yu 888 varieties have no effect on POD activities; however, the variety Azam significantly decreases POD and CAT activities. The ln*RR*_++_ of GPX activity was significantly higher in the Ambrozja variety with chilling stress in the growth chamber study (Figure 5b). The regression analysis revealed that the ln*RR*_++_ of SOD, POD and CAT activities are polynomially affected by melatonin levels. No significant relationship was found between ln*RR*_++_ of APX activity (Figure 3d). These activities are polynomially increased with increasing melatonin level up to 100 µM, and further increasing melatonin application-level declines the SOD, POD and CAT activities. Furthermore, about 17, 26 and 45% of the variability in ln*RR*_++_ of SOD, POD and CAT activities were explained by melatonin application levels (Figure 3b).

### 3.4. Exogenous Melatonin Effect Proline and Reduced the Malonaldehyde, Superoxide, and Hydrogen Peroxide

**Figure 6:**
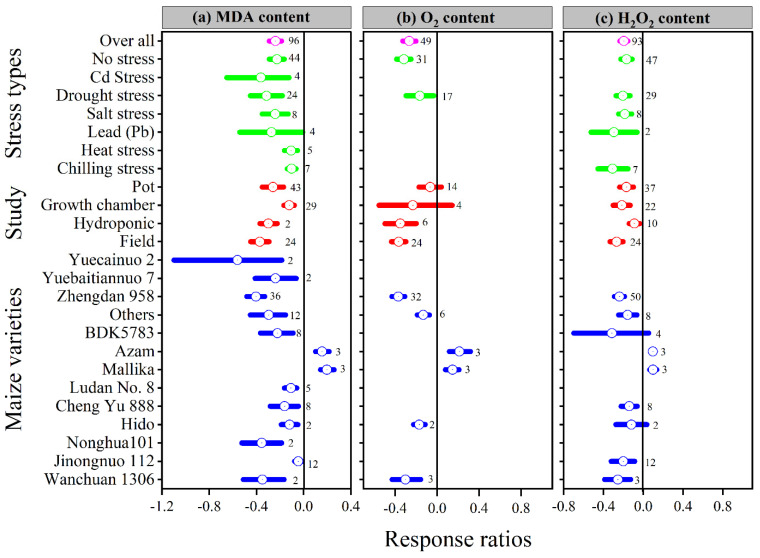
Response ratio of melatonin compared to control with bootstrapped 95% confidence interval on MDA (**a**), O_2_ (**b**) and H_2_O_2_ (**c**), for different stress types, study types, and maize varieties. The vertical line (ln*RR*_++_ = 0) indicates no difference between melatonin and control. Numbers following the box indicate the number of observations for comparison.

Melatonin application had a significant effect on the ln*RR*_++_ of proline, malonaldehyde, superoxide, and hydrogen peroxide under different stress types, study types, and maize varieties (Figure 5a and Figure 6a–c). Compared with the control, melatonin application had no significant effect on the overall ln*RR*_++_ of proline content (Figure 5c). However, significantly decreased the overall ln*RR*_++_ of MDA by 24%, O_2_ by 27% and H_2_O_2_ content by 19% (Figure 6a–c). The results of the current meta-analysis revealed that the melatonin application significantly increased proline by 12% in no stress compared to control. Similarly, among different maize varieties, Nonghua 101 and Wanchuan 1306 increased proline by 24 and 25%, respectively (Figure 5c). Generally, melatonin application mitigated the MDA, O_2_ and H_2_O_2_ content, and the decrease was significant for all types of stresses (Figure 6a–c). Moreover, the ln*RR*_++_ of O_2_ content to melatonin application was not significantly affected with pot and growth chamber studies. Among the maize varieties, Azam and Mallika significantly increased the MDA, O_2_ and H_2_O_2_ content, vice versa. However, melatonin application had no significant effect on H_2_O_2_ content when applied to BDK5783, and Hido maize varieties (Figure 6c). The ln*RR*_++_ of H_2_O_2_ content was not related to melatonin levels, while MDA (R^2^ = 0.15, *p* = 0.02) and O_2_ (R^2^ = 0.47, *p* = 0.02) content decreased linearly with increasing melatonin application levels (Figure 3c). 

### 3.5. Exogenous Melatonin Stimulated Soluble Sugar and Protein, and Reduced Electrolyte Leakage

**Figure 7:**
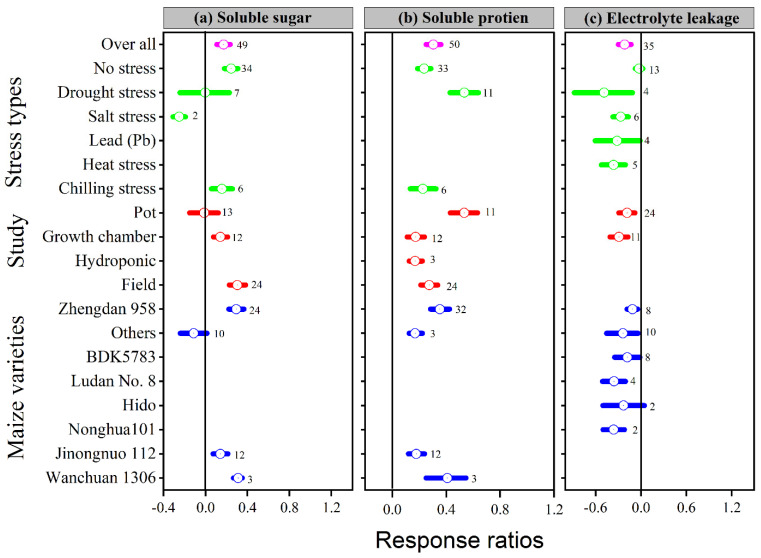
Response ratio of melatonin compared to control with bootstrapped 95% confidence interval on soluble sugar (**a**), soluble protein (**b**), and electrolyte leakage (**c**), for different stress types, study types, and maize varieties. The vertical line (ln*RR*_++_ = 0) indicates no difference between melatonin and control. Numbers following the box indicate the number of observations for comparison.

The ln*RR*_++_ of soluble sugar, soluble protein, and electrolyte leakage to melatonin application significantly differed among different stress types, study types, and maize varieties (Figure 7a–c). Overall, melatonin application significantly stimulated soluble sugar by 17%; *p* < 0.01, with a significantly higher increase of 24, 30 and 31% in the no stress, field study, and Wanchuan 1306, respectively (Figure 7a). Among the stress types, salt stress resulted in lower soluble sugar; in contrast, no stress and chilling stress increased the soluble sugar of the maize crop, whereas the pot study had no significant effect on soluble sugar, it significantly increased within the field study followed by growth chamber study. Similarly, melatonin application also increased soluble protein by 30%; *p* < 0.01, with a significantly higher increase of 54, 54 and 43% in the drought stress, pot study, and Wanchuan 1306 variety, respectively (Figure 7b). The categorical analysis of soluble protein was highly significant in stress types (*p* = 0.001), study types (*p* = 0.001), and maize varieties (*p* = 0.02). These results further demonstrated that the ln*RR*_++_ of drought stress was significantly higher than other stress types in the pot study with the Wanchuan 1306 maize variety and Zhengdan 958. The overall ln*RR*_++_ of electro leakage was significantly decreased with melatonin application compared to control (Figure 7c). Furthermore, the categorical analysis suggested that stress types significantly affected electrolyte leakage (*p* = 0.007), but study types and maize varieties decreased the electro leakage compared to control. The proline content was significantly and linearly increased with increasing melatonin application levels; however, the ln*RR*_++_ of soluble sugar, soluble protein, and relative electrolyte leakage were not significant with application of melatonin concentrations (Figure 3d).

### 3.6. Exogenous Melatonin Impacts Gas Exchange Parameters

The photosynthetic pigments were significantly affected with melatonin application under different stress types, study types, and maize varieties (Table 1). The overall ln*RR*_++_ of photosynthetic rate (*p* = 0.001), stomatal conductance (*p* = 0.001), and transpiration rate (*p* = 0.001) were positively affected by melatonin application. In contrast, the overall and categorical ln*RR*_++_ of intercellular CO_2_ concentration (*p* > 0.05) was not significantly affected by melatonin application, these results suggest that the lower CI is negative (−0.0285) and the upper CI is positive (0.1324), which means the ln*RR*_++_ of intercellular CO_2_ concentration overlaps the zero line. According to our categorical analysis, photosynthetic rate, stomatal conductance, and transpiration rate were positively increased by melatonin application compared to control; however, hydroponic studies have no effect on photosynthetic rate (Table 1). Moreover, this positive increase in photosynthetic rate, stomatal conductance, and transpiration rate was not statistically different among the different study types. The categorical analysis for maize varieties showed that Azam variety significantly decreased the photosynthetic rate and stomatal conductance (*p* < 0.05), however, had no effect on transpiration rate. The relationship between stomatal conductance and transpiration rate was found to be significant for melatonin levels. The stomatal conductance and transpiration rate were polynomially increased and showed 31 and 37% variability, respectively (Figure 3e). The stomatal conductance (R^2^ = 0.31, *p* < 0.01) and transpiration rate (R^2^ = 0.37, *p* < 0.01) were maximized at 100 µM melatonin application.

### 3.7. Exogenous Melatonin Improved Leaf Chlorophyll Content

Table 2 shows that leaf chlorophyll and carotenoid content, demonstrating that melatonin application significantly increased the overall ln*RR*_++_ of total chlorophyll, chlorophyll a and b, and carotenoid compared to control. In addition, most of the stress types resulted in positive and greater leaf chlorophyll content with melatonin application than without melatonin. However, this positive increase was not statistically different among the stress types (*p* > 0.05). Both study types (*p* = 0.001) and maize varieties (*p* = 0.002) significantly increased the leaf chlorophyll content, with a greater increase of 28% in the field study and 59% when using the Wanchuan 1306 maize variety (Table 2). With melatonin application, total chlorophyll, chlorophyll a and b, and carotenoid levels were significantly increased and significantly different among different stress types. Furthermore, when compared to the control, melatonin application into different study types and maize varieties resulted in higher chlorophyll a, b, and carotenoid levels, but these levels were not significantly different in the corresponding groups (study types and maize varieties). The ln*RR*_++_ of chlorophyll *b* (R^2^ = 0.41, *p* < 0.05) and carotenoid (R^2^ = 0.47, *p* < 0.05) increased polynomially with increased melatonin application levels (Figure 3f). 

### 3.8. Effect of Exogenous Melatonin on Leaf Relative Water Content, Leaf Relative Water Potential, and Water Use Efficiency

The leaf relative water content is shown in Table 3, these results confirm that melatonin application significantly increased the leaf relative content compared to control. Among the different stress types, drought stress had a higher leaf relative content (11.4%), which is statistically similar to salt stress (11.1%) and significantly higher than no stress (*p* = 0.002; Table 3). Compared to control, melatonin application resulted in higher ln*RR*_++_ for both pot and growth chamber studies, but both of the study types are statistically similar (*p* > 0.05). Leaf relative water content was also significantly affected by maize varieties. Our categorical analysis revealed that Wanchuan 1306 significantly decreased the leaf relative water content (*p* = 0.02); however, the rest of the varieties resulted in higher leaf relative water content Table 3. The overall ln*RR*_++_ of leaf water potential and water use efficiency were not affected by melatonin application (Table 3). The leaf water potential was also significantly affected by stress types (*p* = 0.03), but not by study types and maize varieties (*p* > 0.05). Lead stress significantly increased leaf water potential compared to other stress in the group. The categorical analysis for water use efficiency revealed that there is no significant effect among the stress types, study types, and maize varieties (*p* > 0.05; Table 3).

## 4. Discussion

It is well known that drought, salt, heavy metals, chilling, and heat stress are the major environmental factors in the soil that inhibit and negatively affect plant growth, development, and production [11,49]. Under stressful situations, plants commonly respond by reducing biomass production, photosynthetic efficiency, enzymatic activity, and producing more ROS [50]. Melatonin is a newly discovered plant growth regulator that has a remarkable growth-promoting influence at all stages of plant development, from seed germination to vegetative and reproductive development [51,52,53]. This meta-analysis evaluated the protective effects of melatonin application on different maize varieties and stress types. Our meta-analysis showed that melatonin had a positive effect on plant growth parameters compared to control, which confirmed our first hypothesis that melatonin significantly increased plant height, leaf area, root length, fresh and dry root weight, and fresh and shoot weight. The increase in the above parameters with melatonin was probably due to its remarkable antioxidant effects against oxidative stress, and it is involved in numerous physiological systems in plants [1,52]. Our meta-analysis showed that the melatonin application significantly increased the morphological attributes of maize by modulating its plant height, leaf area, root length, fresh and dry root weight, and fresh and dry shoot weight compared to control (Figure 1 and Figure 2). Recently, it was noted that compared to control, melatonin application at 100 µM had significantly greater root and shoot biomass and root length of tobacco under Cd stress [54], and greater root and shoot biomass, leaf area, root and shoot length, and primary root length of maize crop were reported under drought stress [1,55]. In addition, the melatonin had a strong and positive effect on the ln*RR*_++_ of plant height, leaf area, fresh and dry root weight, fresh and dry shoot weight under drought and salt stress, root length under chilling stress, and root diameter under salt stress. Similarly, melatonin application increased plant height and leaf area for all maize varieties, but for Yuecainuo 2 and Wanchuan 1306, the melatonin application resulted in short root length, and Wanchuan 1306 had thinner root diameter, lower dry root and shoot weight (Figure 1 and Figure 2). Conversely, stress-reduced root length, plant height, leaf surface area, and chlorophyll content while increasing ROS such as O_2_ and H_2_O_2_, as well as oxidative damage such as MDA content and electrolyte leakage level. However, melatonin application significantly increased plant growth parameters while reducing ROS content and oxidative damage compared to control [1,10].

Melatonin has also been shown to boost antioxidant enzyme activities by maintaining higher antioxidant enzyme activity to reduce oxidation damage in plants caused by abiotic stress, such as cold, drought, Cd, lead, heat, and salinity [1,56,57]. Antioxidant enzyme activity regulation is an intrinsic plant response to counteract oxidative stress generated by diverse biotic and abiotic environmental factors [33,58]. The overall positive ln*RR*_++_ of SOD, POD, CAT, and APX revealed that melatonin application had a significant effect on the antioxidant defense systems of the maize crop compared with control but had no effect on GPX activity (*p* < 0.05; Figure 4 and Figure 5). The melatonin application significantly increased the SOD activity of maize leaf under drought, salt, and chilling stress, but had no effect under Cd, lead, and heat stress. In addition, a significant positive effect of melatonin on POD and CAT activities under all stress types was detected, except that Cd stress had no effect on SOD and significantly decreased CAT activity compared to control (Figure 4b,c). Likewise, melatonin application to different maize varieties had effects on the antioxidant defense system, suggesting that Yuecainuo 2 and Azam significantly decreased SOD activity, Azam decreased POD and APX activities, and Yuecainuo 2, Yuebaitiannuo 7 and Azam had lower CAT activity. Melatonin application drastically decreased oxidative damage by modulating antioxidant activities such as SOD, POD, CAT, and APX activities, resulting in lower MDA content in wheat, maize, and Bermuda grass [1,59,60]. Our findings are in agreement with the previous findings of Lin et al. [61], who reported that increasing melatonin application up to 100 µM improved antioxidant enzyme activities, but further increasing melatonin from 150 to 200 µM had a deleterious impact on Tamarillo (tree tomato). In contrast, Ma, Huang, Li, Ashraf, Yang, Liu, Xu, Li and Mo [1] detected that melatonin application at 200 μM could increase the antioxidant defense mechanism in maize. Thus, melatonin application at lower rates could be ascribed to a modulation in antioxidant activities, which can also result in a significant reduction in oxidative plant damage [1,62]. In order to protect cells from damage, the antioxidative defense system includes SOD, POD, CAT and APX. This system helps to maintain the equilibrium of ROS in cells, slows down membrane lipid peroxidation, and resists stress [31]. Melatonin can directly scavenge ROS, maintain steady H_2_O_2_ concentrations [63], and indirectly scavenge H_2_O_2_ by stimulating CAT and POD activities in plants under environmental stress conditions [31].

The results of this meta-analysis showed that the melatonin application significantly decreased the overall ln*RR*_++_ of MDA, O_2_, H_2_O_2_, and electrolyte leakage by 24, 27, 19 and 21% compared to control (Figure 6a–c and Figure 7c). Additionally, the toxicity of Pb damaged membrane integrity, resulting in enhanced membrane deterioration and the formation of ROS, such as H_2_O_2_ and O_2_, in plant leaves [8,64]. Heavy metals and environmental stress are the key issues that disturb virtually all features of the biochemistry and physiology of plants. Okant and Kaya [8] reported that the universal antioxidant melatonin is able to easily pass through the plasma membrane and go into the subcellular sections, and thus the use of melatonin in plants under stress could be justified. Furthermore, the melatonin supplementation reduced Pb-induced oxidative stress by lowering H_2_O_2_ and MDA levels and lowering the electrolyte leakage rate [65]. Melatonin application significantly decreases the accumulation of H_2_O_2_, O_2_, and MDA in maize under drought stress [66] and cucumber seedlings under salinity stress [67]. Likewise, our results also suggest that melatonin application to all kinds of stress significantly decreases MDA, O_2_, H_2_O_2_ and electrolyte leakage compared to control (*p* < 0.05; Figure 6a–c and Figure 7c). It has been demonstrated that exogenous melatonin has a protective effect against membrane damage when subjected to salt stress [31] and Cd stress [1], owing to a decrease in H_2_O_2_ and MDA levels in leaves. Guo, Li, Zhao, Xue and Zhang [2] demonstrated that MDA levels increased under drought stress; however, melatonin application reduced the accumulation of H_2_O_2_, O_2_, and MDA levels in both cultivars SD609 and SD902 under drought stress. Increased SOD, POD, and APX activities were found to be associated with lower electrolyte leakage and MDA concentration in melatonin-treated maize leaves [24], and cucumber leaves [56], under salt stress, showing that salinity-induced oxidative damage is susceptible to mitigation through the application of melatonin. Under salinity stress, plants enhance their osmotic adaptation capacity by synthesizing and accumulating organic osmolytes; soluble sugar is one of the essential osmolytes for osmotic adjustment [39,68]. The categorical analysis revealed that the soluble sugar was dramatically increased by melatonin application under no and chilling stress, and protein under all stress types, but salt stress resulted in lower soluble sugar than control (Figure 7a,b). It has been demonstrated that drought stress can affect protein production [69]. However, melatonin has been found to repair PSII by maintaining protein availability in tomato under salt stress [70], and to protect PSII proteins in maize under drought stress [71].

Reductions in photosynthetic pigments and antioxidant enzyme activities, as well as morphological growth, were found in maize exposed to salt stress [24], Cd stress [1,6], and drought stress [21]. Drought stress triggers stomatal closure or destruction of photosynthetic reaction in plants, which can result in a significant decrease in photosynthetic rate [72,73]. Previous studies demonstrated that melatonin application had significantly higher photosynthetic efficiency, transpiration rate, and stomatal conductance than control in tomato [70], wheat [74] and Medicago sativa [75]. Similarly, our results showed that the overall ln*RR*_++_ of melatonin-treated maize resulted in a higher photosynthetic rate (17%), stomatal conductance (17%), and transpiration rate (21%) than those of the control (Table 1). However, melatonin had a nonsignificant effect on the overall and categorical ln*RR*_++_ of intercellular CO_2_ concentration. Furthermore, melatonin application to drought and salt stress significantly increased the photosynthetic rates, stomatal conductance, and transpiratory rates compared to control. In contrast, the negative bootstrap value suggests that the melatonin application to no-stress maize is not statistically significant (Table 1). Campos, Avila, de Souza, Azevedo and Alves [73] demonstrated that enhanced tomato tolerance to drought stress by stimulating cuticle production, which in turn reduced water loss. In drought stress conditions, those plants treated with 300 M of melatonin showed higher stomatal conductance and photosynthetic and transpiration rates, providing a greater supply of assimilates for growing tissues. A previous study reported that melatonin treatment enhanced gas exchange parameters and thus biochemical reactions by regulating leaf water potential [73]. Previous studies have shown that tomato seedlings treated with melatonin provide greater stomatal conductance and contribute to the maintenance of photosynthetic rates under water deficit conditions [70], Arabian coffee [73], and maize under salt stress conditions [15]. 

Melatonin regulates flowering, photosynthesis, chlorophyll synthesis, callus formation, root regeneration, and plant photosynthetic properties under varied environmental stressors. [56,76]. Melatonin is also an antioxidant that protects plants from biotic and abiotic stresses [38,77], such as salt [41,78], drought [79,80,81], cold [59,82], and heavy metal stress [7]. Melatonin application reduced stress-induced photosynthetic inhibition (Table 2), suggesting that the overall ln*RR*_++_ of total chlorophyll, chlorophyll *a* and *b*, and carotenoid were 18, 38, 24, and 18% higher than control, respectively. These results are in line with the findings of Zhou, Zhao, Cao, Hu, Du, Baluška and Zou [70] and Chen, Mao, Sun, Huang, Ding, Gu, Liao, Hu, Zhang and Yuan [3], they found that the increased biomass in melatonin-treated plants could be attributed to the plants’ ability to retain a high photosynthetic capacity. However, melatonin had no effect on total chlorophyll under Cu stress, chlorophyll *a* and *b* under no stress, and carotenoid under no and salt stress conditions (Table 2). Melatonin at the rate of 10 µM was reported to improve nitrogen metabolism and proline stability in drought-stressed alfalfa, resulting in greater levels of chlorophyll content [75,83]. It is known that salt stress can limit the synthesis of chlorophyll and speed up its decomposition because of chlorophyll fragility and susceptibility to ROS [7,84]. There is some evidence that melatonin treatment delays leaf senescence and improves tolerance to salinity stress in rice leaves [85], as well as improving the photosynthetic pigment-synthesis pathway and slowing the decomposition rate of chlorophyll in maize leaves under salinity stress [31]. The overall relative water content in the leaf was significantly increased with melatonin under stress conditions compared to control, but melatonin application had no effect on relative water content under no stress conditions (Table 2). These results are supported by Qiao, Ren, Yin, Liu, Deng, Liu and Wang [21], who demonstrated that under control conditions, whether with or without melatonin application, the leaf relative water content did not alter, although melatonin effectively reduced the stress-induced reduction in leaf relative water content. According to Jiang, Li and Song [24], who reported that plants exposed to salinity stress had lower leaf water content, this was alleviated by 1 µM melatonin application. The categorical meta-analysis revealed that the melatonin application had no significant effect on leaf water potential under no stress, drought, and salt stress conditions. However, lead stress resulted in 11% higher water potential compared to control (Table 2). In contrast, a recent study reported that leaf relative water potential significantly increased by 15.9% in melatonin-treated plants under drought stress conditions compared to control [21]. Sharma and Dubey [86] and Okant and Kaya [8] reported that lead toxicity disrupted water status in plants, which could be an explanation of low water potential under Pb stress. Melatonin application resulted in higher leaf water potential than those of the water deficit [73].

## 5. Conclusions

The meta-analysis identified that melatonin has a varied effect on plant growth, antioxidant defense system, gas exchange parameters, photosynthetic pigments, and soluble sugar and protein content under different stress types, study types, and maize varieties. Melatonin application increased plant growth, antioxidant enzyme activities, gas exchange parameters, photosynthetic pigments, and soluble sugar and protein compared to control. The greater Q_T_ and I-square values showed the total heterogeneity among the studies in terms of stress types, study types, and maize varieties. Drought stress with melatonin had the highest photosynthetic rate and stomatal conductance followed by salt stress, but the transpiration rate was greater in salt stress. Melatonin had no effect on the overall and category ln*RR*_++_ for intercellular CO_2_ concentration. However, melatonin had an overall negative impact on MDA, O_2_, H_2_O_2_ and electrolyte leakage under all stress types, but no stress had any effect on electrolyte leakage compared to control. The categorical analysis showed that all the maize varieties decreased the MDA, O_2_ and H_2_O_2_ content but increased with Azam and Mallika, whereas BDK5783 and Hido had no effect on H_2_O_2_ content. We concluded from our results that melatonin performs various functions under stress and that tailored changes can increase maize’s stress tolerance through decreasing ROS and improving the antioxidant defense system. However, further research is needed to determine the effect of melatonin in combination with synthetic fertilizers and other signaling molecules in response to various biotic and abiotic stress, as well as the optimal dose of melatonin.

## Figures and Tables

**Table 1:** Response ratio of melatonin compared to control with bootstrapped 95% confidence interval on photosynthetic rate, stomatal conductance, transpiration rate, and intercellular CO_2_ concentration for different stress types, study types, and maize varieties. The negative bootstrap values indicate no difference between melatonin and control.

Variables	Photosynthetic Rate				Stomatal Conductance				Transpiration Rate				Intercellular CO_2_ Concentration			
	ln*RR*_++_	Bootstrap CI		N	ln*RR*_++_	Bootstrap CI		N	ln*RR*_++_	Bootstrap CI		N	ln*RR*_++_	Bootstrap CI		N
Overall	0.1731	0.1340	0.2120	95	0.1703	0.1231	0.2187	83	0.2145	0.1596	0.2701	63	−0.0240	−0.0834	0.0389	12
Stress types																
No stress	0.0957	0.0607	0.1310	54	0.0263	−0.0330	0.0798	33	0.0194	−0.0034	0.0465	22	−0.0085	−0.1013	0.0725	5
Drought stress	0.2705	0.1919	0.3599	33	0.2627	0.1843	0.3460	33	0.3167	0.2404	0.4028	33	−0.0230	−0.0727	0.0311	4
Salt stress	0.2666	0.1893	0.3910	8	0.2618	0.2183	0.3058	17	0.3556	0.2689	0.4436	8	−0.0573	−0.1979	0.3435	3
Study types																
Pot	0.1822	0.1230	0.2478	53	0.1788	0.1258	0.2338	65	0.2225	0.1546	0.2953	45	0.0097	−0.0419	0.0530	6
Growth chamber	0.1325	0.0494	0.2194	8	0.1516	0.0540	0.2462	8	0.1382	0.0319	0.2574	8	−0.0663	−0.1657	0.0428	4
Hydroponic	0.1274	−0.0177	0.3193	10	0.135	0.0402	0.2398	10	0.2598	0.1079	0.4218	10	−0.0683	−0.1878	0.3435	2
Field	0.1879	0.1500	0.2247	24												
Maize varieties																
Zhengdan 958	0.1949	0.1595	0.2337	50	0.1858	0.1340	0.2431	26	0.2522	0.1728	0.3292	26				
Others	0.2079	0.0590	0.3664	20	0.1841	0.0365	0.3329	20	0.3290	0.1598	0.5327	12	−0.0021	−0.0769	0.0742	8
Azam	−0.1401	−0.1643	−0.0953	3	−0.1539	−0.2029	−0.1076	3	−0.0179	−0.0535	0.0001	3				
Mallika	0.1544	0.1014	0.2124	3	0.0342	0.00001	0.0606	3	0.0425	0.0343	0.0477	3				
Cheng Yu 888	0.0800	0.0260	0.1425	14	0.0887	−0.0164	0.1838	14	0.1084	0.0286	0.2024	14	−0.065	−0.1665	0.0402	4
Wanchuan 1306	0.2434	0.1488	0.3461	3	0.2618	0.1476	0.3482	3	0.3408	0.2368	0.4239	3				
Shaanke 9	0.4368	0.0011	0.9240	3	0.3954	0.0124	0.8503	12	0.3490	−0.0115	0.7173	2				
Vega F1					0.2456	0.2094	0.2786	2								

The negative bootstrap values indicate no difference between melatonin and control. The ln*RR*_++_ is the response ratio, and N is the number of observations for comparison. The ln*RR*_++_ is the response ratio, and N is the number of observations for comparison.

**Table 2:** Response ratio of melatonin compared to control with bootstrapped 95% confidence interval on leaf chlorophyll content, chlorophyll *a* content, chlorophyll *b* content, and carotenoid for different stress types, study types, and maize varieties.

Variables	Leaf Chlorophyll Content				Chlorophyll a Content				Chlorophyll b Content				Carotenoid			
	ln*RR*_++_	Bootstrap CI		N	ln*RR*_++_	Bootstrap CI		N	ln*RR*_++_	Bootstrap CI		N	ln*RR*_++_	Bootstrap CI		N
Overall	0.1800	0.1352	0.2210	83	0.3826	0.2796	0.5030	29	0.2400	0.1567	0.3251	29	0.1813	0.0925	0.2619	24
Stress types																
No stress	0.1729	0.1301	0.2190	52	0.0382	−0.0172	0.1237	9	0.0215	−0.0402	0.1042	9	0.0050	−0.0455	0.0755	6
Drought stress	0.3080	0.0980	0.4846	11	0.6257	0.4711	0.7673	12	0.4087	0.2909	0.5199	12	0.2802	0.1685	0.3910	15
Salt stress	0.1883	0.0907	0.3045	4	0.5198	0.3970	0.6408	4	0.3250	0.2397	0.3785	4	0.0805	−0.0336	0.1587	3
Lead (Pb)	0.1149	0.0931	0.1366	2	0.3465	0.1421	0.5422	4	0.1707	0.0321	0.3049	4				
Chilling stress	0.1870	0.1106	0.3503	8												
Cu stress	−0.0165	−0.1522	0.0797	6												
Study types																
Pot	0.1386	0.0567	0.2287	28	0.4167	0.2974	0.5447	25	0.2424	0.1488	0.3298	25	0.1886	0.0936	0.2792	22
Growth chamber	0.0754	0.0130	0.1333	24												
Hydroponic	0.1935	0.1084	0.2680	7	0.2077	0.0869	0.3348	4	0.2328	0.0611	0.3438	4	0.0212	0.0134	0.0290	2
Field	0.2757	0.2218	0.3285	24												
Maize varieties																
Zhengdan 958	0.2640	0.2092	0.3107	26	0.4433	0.2728	0.6132	16	0.2773	0.1512	0.4092	16	0.2086	0.0989	0.3178	16
Others	0.1099	0.0558	0.1686	48	0.3265	0.2944	0.3603	2	0.3371	0.3083	0.3683	2	−0.0352	−0.1792	0.0651	5
BDK5783	0.0683	0.0229	0.1149	4	0.1888	0.0531	0.3470	8	0.0968	0.0217	0.1906	8				
Wanchuan 1306	0.5892	0.4276	0.7353	3	0.6506	0.4892	0.7857	3	0.3666	0.2020	0.5048	3	0.3072	0.1951	0.4978	3

The negative bootstrap values indicate no difference between melatonin and control. The ln*RR*_++_ is the response ratio, and N is the number of observations for comparison.

**Table 3:** Response ratio of melatonin compared to control with bootstrapped 95% confidence interval on relative water content, leaf water potential, and water use efficiency for different stress types, study types, and maize varieties.

Variables	Relative Water Content				Leaf Water Potential				Water Use Efficiency			
	Ln*RR*_++_	Bootstrap CI		N	Ln*RR*_++_	Bootstrap CI		N	Ln*RR*_++_	Bootstrap CI		N
Overall	0.0699	0.0391	0.1025	60	0.0016	−0.0405	0.0449	14	−0.024	−0.0834	0.0389	12
Stress types												
No stress	−0.0023	−0.0126	0.0066	22	0.0067	−0.0296	0.0301	8	−0.0085	−0.1013	0.0725	5
Drought stress	0.1144	0.0537	0.1825	28	−0.1116	−0.1765	−0.0603	2	−0.023	−0.0727	0.0311	4
Salt stress	0.111	0.0823	0.1459	9	−0.0169	−0.0545	0.0548	2	−0.0573	−0.1979	0.3435	3
Lead (Pb)					0.1149	0.0931	0.1366	2				
Study types												
Pot	0.0822	0.0377	0.1351	40	0.0208	−0.0262	0.0676	8	0.0097	−0.0419	0.053	6
Growth chamber	0.0456	0.0252	0.0662	20	−0.0364	−0.1166	0.0325	6	−0.0663	−0.1657	0.0428	4
Hydroponic									−0.0683	−0.1878	0.3435	2
Maize varieties												
Zhengdan 958	0.091	0.0514	0.1303	24	−0.0731	−0.1765	0.0337	2				
Others	0.1267	0.0345	0.2494	15					−0.0021	−0.0769	0.0742	8
BDK5783					0.0673	0.0229	0.1203	4				
Cheng Yu 888	0.0246	0.0031	0.0477	14	−0.0179	−0.0557	0.0185	8	−0.065	−0.1665	0.0402	4
Nonghua101	0.0534	0.0283	0.0784	2								
Wanchuan 1306	−0.1276	−0.1865	−0.0796	3								
Hido	0.0434	0.0111	0.0746	2								

The negative bootstrap values indicate no difference between melatonin and control. The ln*RR*_++_ is the response ratio, and N is the number of observations for comparison.

## Data Availability

The datasets used and/or analyzed during the current study will be available from the corresponding author on reasonable request.

## Supplementary Materials

The following supporting information can be downloaded at: https://www.mdpi.com/article/10.3390/antiox11030512/s1, Figure S1: Kernel density estimates (smoothed version of the histogram) for plant growth parameters; Figure S2: Kernel density estimates (smoothed version of the histogram) for gas exchange parameters, and chlorophyll pigments; Figure S3: Kernel density estimates (smoothed version of the histogram) for antioxidant enzymes, soluble sugar, soluble protein, proline, and relative electro leakage; Table S1 Observations’ number (n) and results of testing publication bias and heterogeneity for each target variable;
