# Supplementary material for: Melatonin Application Alleviates Stress-Induced Photosynthetic Inhibition and Oxidative Damage by Regulating Antioxidant Defense System of Maize: A Meta-Analysis
Source: Antioxidants (Basel). 2022 Mar 8;11(3):512. doi: 10.3390/antiox11030512 (PMC8944576; doi:10.3390/antiox11030512)
Supplement: Supplementary file 1 [file antioxidants-11-00512-s001.zip › antioxidants-1616745-supplementary.pdf]

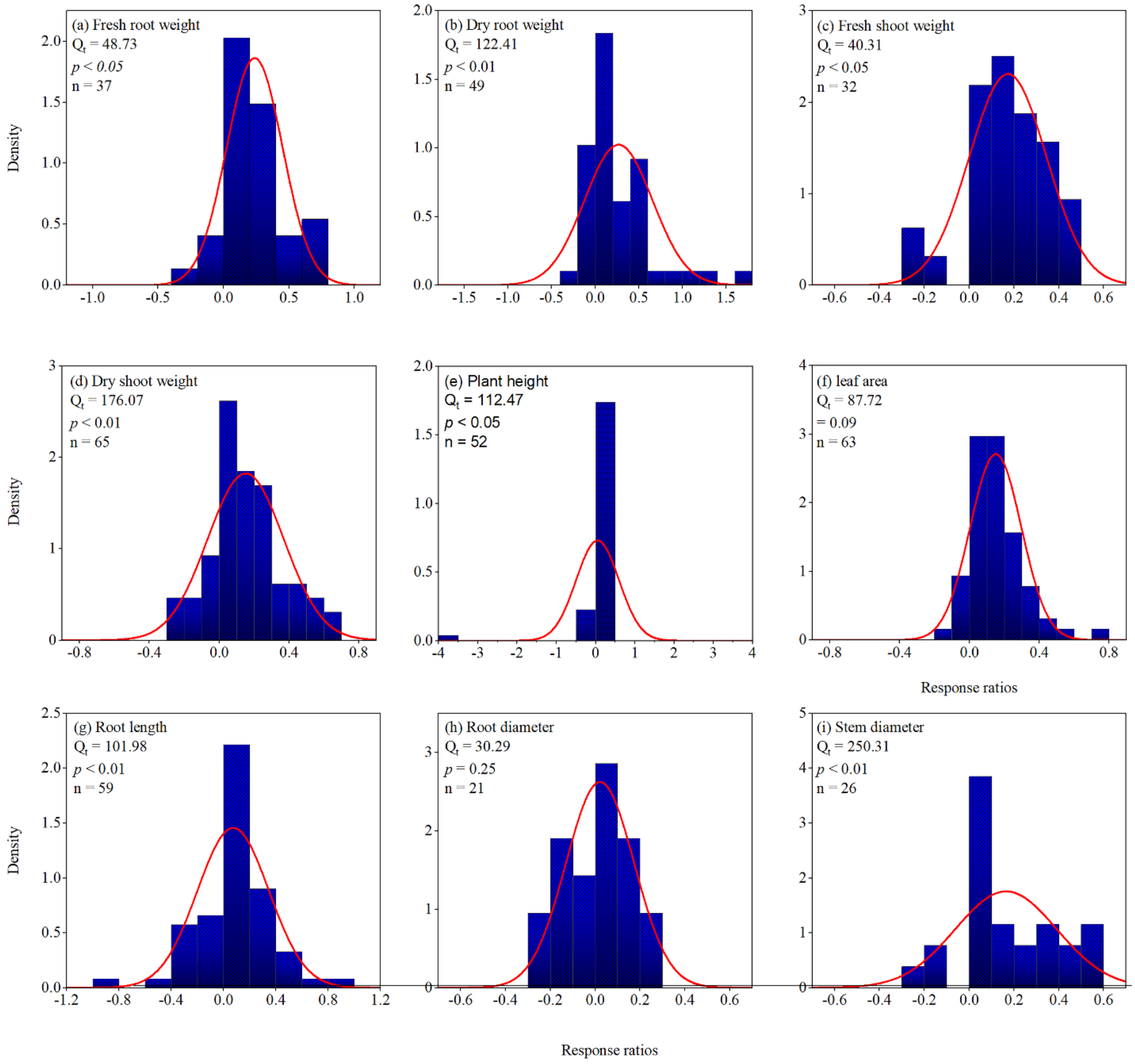

Fig.

Figure S1. Kernel density estimates (smoothed version of the histogram) for plant growth parameters. The red curves were fitted by the Gaussian function using the software Origin 2021. Q_t is among studies heterogeneity for all observations in the meta-analysis and n is the number of paired observations.

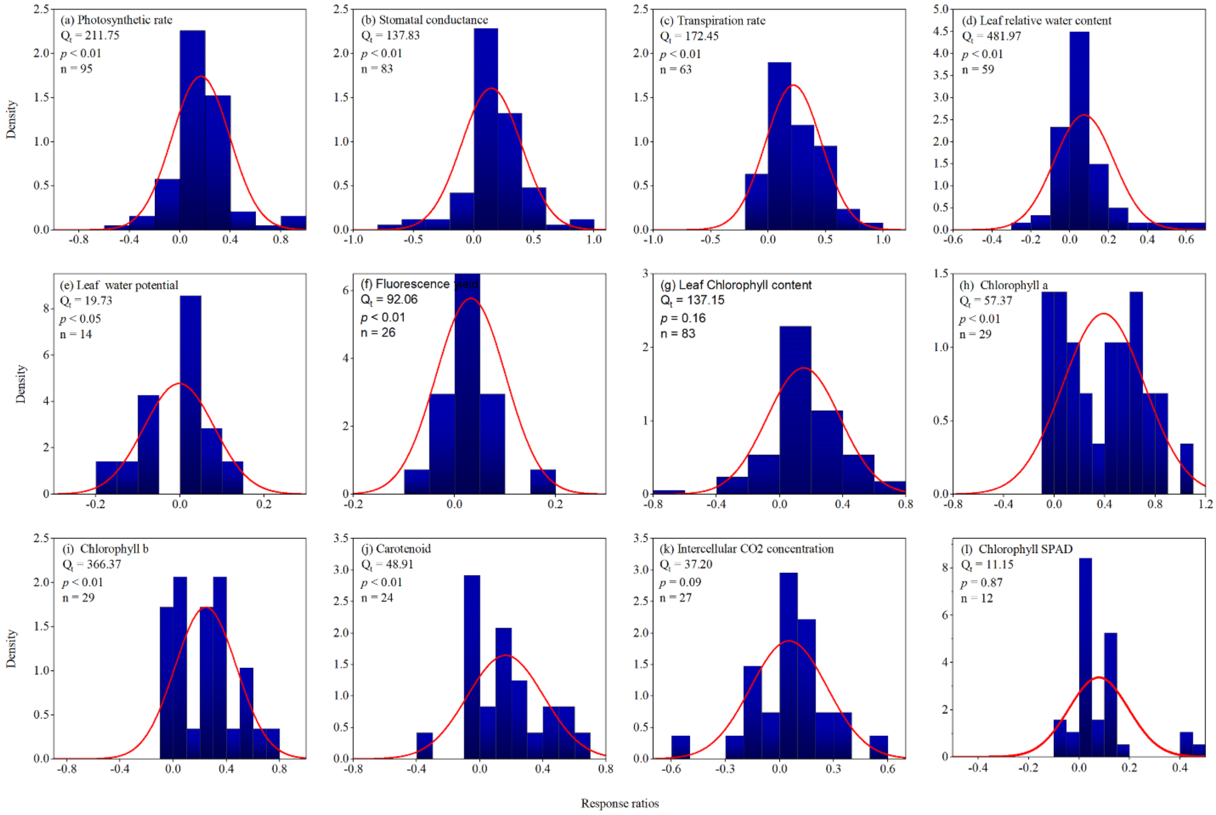

Fig.

Figure S2. Kernel density estimates (smoothed version of the histogram) for gas exchange parameters, and chlorophyll pigments. The red curves were fitted by the Gaussian function using the software Origin 2021. Q_t is among studies heterogeneity for all observations in the meta-analysis and n is the number of paired observations.

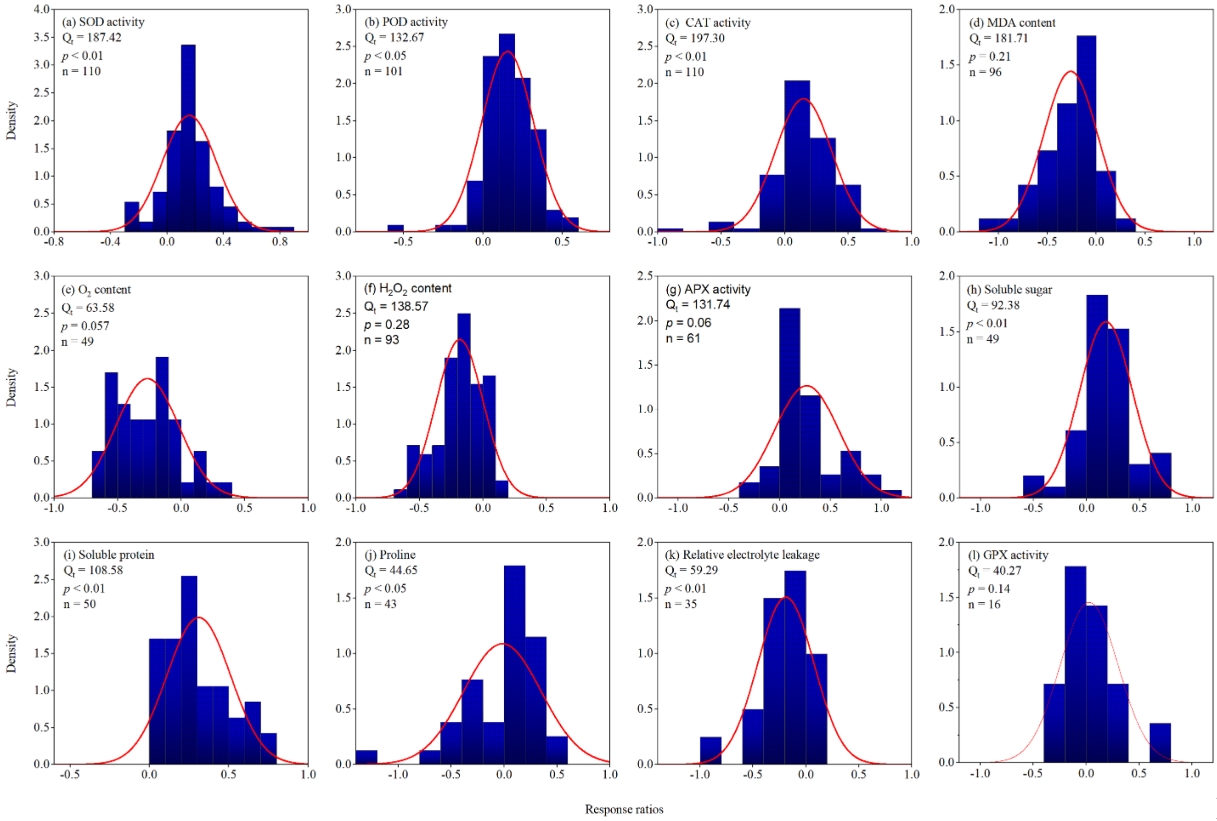

Fig.

Figure S3. Kernel density estimates (smoothed version of the histogram) for antioxidant enzymes, soluble sugar, soluble protein, proline, and relative electro leakage. The red curves were fitted by the Gaussian function using the software Origin 2021. Q_t is among studies heterogeneity for all observations in the meta-analysis and n is the number of paired observations.

Table S1. Observations' number (n) and results of testing publication bias and heterogeneity for each target variable.

Moderator	Variables	Number of Observations (n)	Fail-Safe Number (N)	*p*-Value for Rank-Order Correlation	Percent I-Square (%I^2)
Growth parameter	Fresh root weight	37	23615	0.1590	135
	Dry root weight	49	25879	0.0519	255
	Fresh shoot weight	32	41993	0.1410	130
	Dry shoot weight	65	41058	0.7055	275
	Plant height	52	30788	0.1673	221
	Leaf area	63	61699	0.4359	142
	Root length	59	14782	0.2882	176
	Root diameter	21	216	0.6658	151
Gas exchange parameters	Photosynthetic rate	95	48264	0.0538	225
	Stomatal conductance	83	66551	0.0061	168
	Transpiration rate	63	45573	0.0852	278
	Intercellular CO_2 concentration	27	748	0.3647	143
Chlorophyll pigments	Total chlorophyll	83	61203	0.0007	167
	Chlorophyll a	29	7370	0.1682	205
	Chlorophyll b	29	6039	0.7803	130
	Carotenoid	24	3998	0.2127	213
Leaf water	Relative water content	60	18560	0.0259	831
Plant enzymes	SOD	110	148417	0.0081	174
	POD	101	73009	0.0004	133
	CAT	110	115831	0.4025	183
	APX	61	48188	0.7782	227
	GPX	16	3.7	0.0848	268
ROS	O_2	49	42536	0.4543	135
	H_2O_2	93	91624	0.7422	151
MDA	MDA	96	106138	0.1946	191
Proline	Proline	43	1797	0.6915	106
Soluble sugar	Soluble sugar	49	10497	0.0066	192
Soluble protein	Soluble protein	50	66576	0.0119	222
Electro leakage	Electro leakage	35	38714	0.2057	191
